# Multi-Angle Optical Image Automatic Registration by Combining Point and Line Features

**DOI:** 10.3390/s22030739

**Published:** 2022-01-19

**Authors:** Jia Su, Juntong Meng, Weimin Hou, Rong Wang, Xin Luo

**Affiliations:** 1 School of Information Science and Engineering, Hebei University of Science and Technology, Shijiazhuang 050018, China; sujia@hebust.edu.cn (J.S.); mengjuntong@stu.hebust.edu.cn (J.M.); hwm@hebust.edu.cn (W.H.); 2 Yangtze Delta Region Institute (Huzhou), University of Electronic Science and Technology of China, Huzhou 313001, China; 202121070112@std.uestc.edu.cn; 3 School of Resources and Environment, University of Electronic Science and Technology of China, Chengdu 611731, China

**Keywords:** image registration, scene splicing, line features, LSD, GMM

## Abstract

Image registration is an important basis of image processing, which is of great significance in image mosaicking, target recognition, and change detection. Aiming at the automatic registration problem of multi-angle optical images for ground scenes, a registration method combining point features and line features to register images is proposed. Firstly, the LSD (Line Segment Detector) algorithm is used to extract line features of images. The obtained line segments whose length are less than a given threshold are eliminated by a visual significant algorithm. Then, an affine transform model obtained by estimating a Gaussian mixture model (GMM) is applied to the image to be matched. Lastly, Harris point features are utilized in fine matching to overcome shortages of methods based on line features. In experiments, the proposed algorithm is compared with popular feature-based registration algorithms. The results indicate that the proposed algorithm in this work has obvious advantages in terms of registration accuracy and reliability for optical images acquired at different angles.

## 1. Introduction

The specific task of image registration technology is to determine point-by-point mapping relationships among images acquired from same scenes in different shooting conditions, such as angles, time, and sensors. It can realize the fusion of multi-image information and the expansion of visual ranges. At present, this technique is widely used in automatic driving, target tracking, remote sensing mapping, medical diagnosis, military survey, and other fields [1,2,3]. Recently, there have been many achievements under the efforts of scholars and engineers in the field of optical image registration. The Harris corner algorithm is combined with the scale-invariant feature transform (SIFT) operator for registering satellite-borne optical imagery, such as panchromatic and multispectral images [4]. An adaptive redundant key-point elimination method (RKEM)-SIFT is proposed by Zahra [5]. It reduces computational complexities while improving image matching performances. In addition, due to significant differences in their imaging mechanisms, a rapid and robust method based on SURF was designed for multi-modal image registration by exploiting local edge information [6]. It can simultaneously satisfy requirements of real-time and accuracy. The KAZE algorithm is combined with a modified version of the speeded-up robust features (SURF) descriptor for registering synthetic aperture radar (SAR) images [7]. Wang proposed an improved KAZE-HOG algorithm, which has good capability to resist scale and rotation transforms [8]. Zheng put forward an image registration method based on RANSAC (Random Sample Consensus), which is suitable for processing aerial video. It incorporates prior sampling to possibly generate more correct samples [9]. These methods have their own characteristics, but they are not designed for multi-angle images.

The focus of this work is on automatically registering optical images acquired from different view angles on the ground. Images from limited fields of view can be stitched together by using registration technology so as to broaden visual fields or generate panoramas. However, there are various divergences in images caused by differences of view angles, such as displacement, scale, and rotation. It is essential to find invariant features in images of different view angles for registering and stitching them properly. The remainder of this article is organized as follows. The second section introduces the procedure of our algorithm, in which rough matching by using LSD and GMMs and fine matching based on point features are described in detail. In the third section, the proposed registration method is verified and compared with other algorithms by using multi-angle optical images. Finally, the discussion and conclusion are given in the fourth section.

## 2. Methodology

**Figure 1:**
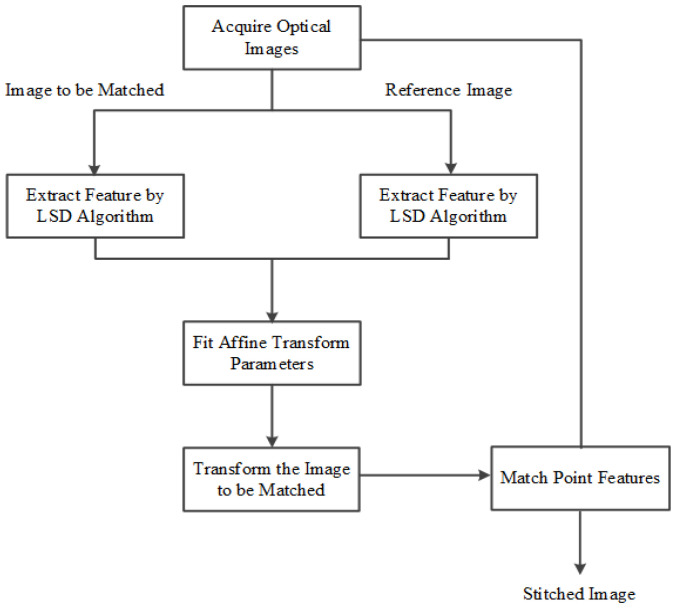
The proposed image registration algorithm flow chart.

For ordinary optical images with certain differences of view angles, point and line features are combined in order to deal with the limitations of popular registration methods based on point features in this work. First of all, the LSD (Line Segment Detector) algorithm is utilized during rough matching and the transform affine model parameters of rough registration are estimated by using line features and GMM (Gaussian Mixture Models). Then, Harris point feature extraction is applied in fine matching to enhance registration accuracy. The procedure of our algorithm is demonstrated in Figure 1.

### 2.1. Rough Matching

#### 2.1.1. Line Detection by LSD

The LSD algorithm is a line detection algorithm proposed by Gioi [10]. It has great advantages in calculation speed. In particular, it is much faster than the Hough transform [11]. Generally, line detection algorithms are based on edge detection, and edge information needs to be computed first. In contrast, LSD directly uses the gray information of images to generate line segmentation matrices without adjusting algorithm parameters [12,13,14]. The LSD algorithm mainly includes three steps: detecting the candidate regions for lines, rectangular approximation of the candidate regions for lines, and line verification.

**Figure 2:**
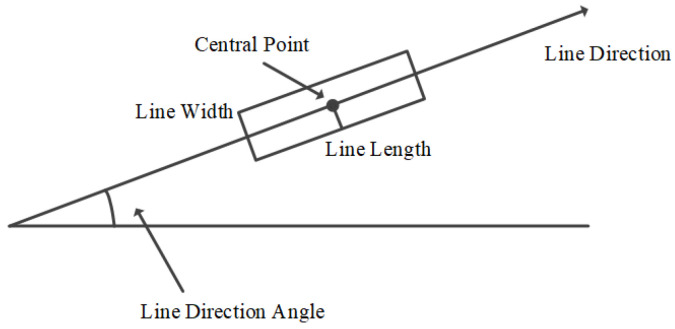
Rectangle estimation for a line segment from a candidate region.

(1) Detect candidate regions for lines. Due to quantization noises in imaging processes, there are sawteeth in the edges of images. If line detection is conducted directly, the extraction results will be affected. Therefore, an optical image is blurred through Gaussian down-sampling to the 80% size of the original image. Then, the gradient at each point is calculated within a region of 2 × 2 pixels. The amplitude of the gradient represents the change degree of gray values in the image. Large values denote large gray differences of neighboring pixels, and small values mean that pixels and their neighboring pixels are very likely to belong to a same region. Hence, in order to effectively extract line regions, the points with small gradient amplitudes will be deleted according to a preset threshold.

(2) Generate candidate regions for lines. The determination of candidate regions for lines is realized by iterations. At the beginning of an iteration, a candidate region is a point at which the gradient amplitude is at its maximum, and the direction of this candidate region is the gradient direction at the point. Next, the gradient at its neighboring points in the candidate region is calculated. When the angle between the gradient direction at a neighboring pixel point and the region direction is less than a preset threshold, the neighboring points are marked as a point belonging to the candidate region. Then, the direction of the candidate region will be updated. However, the candidate region is irregular and cannot be employed to represent a line segment. Therefore, it is necessary to estimate a rectangle for a line according to the region [15]. The specific scheme is demonstrated in Figure 2, including determining the direction, width, height, and center of the estimated rectangle.

(3) Verify lines. It is necessary to verify lines to avoid false detections for as many as possible. The NFA (Number of False Alarms) is chosen as the verification indicator [16]. Consider an image of *M* × *N* pixels, it is defined as follows.

(1) NFA(r)=(MN)52*B(n,k,p)

where *r* represents a candidate region for lines, and *B*(*n*, *k*, *p*) is a binomial model. The *p* is a given precision value for each candidate region. The *n* and *k* stand for the number of pixels contained in this region and the number of points whose direction are identified as that of the region, respectively. The NFA value can be derived by the following formula [17].

(2) NFA(r)=(MN)52*∑j=kn(nk)pj(1-p)n-j

**Figure 3:**
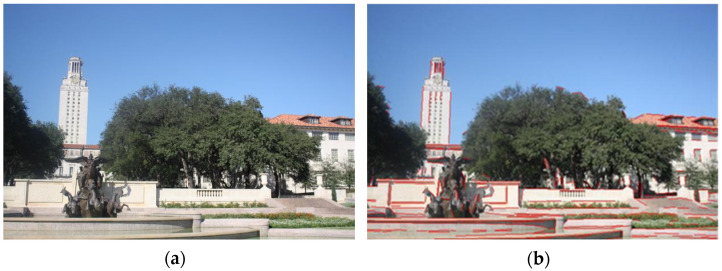
Extracting line features from an image: (**a**) The original image (615 × 410 pixels); (**b**) the line features extracted by LSD.

If the NFA value of a candidate region is less than a given threshold, it is thought that a line is correctly detected. Figure 3 exhibits an example of a line feature extraction by LSD.

#### 2.1.2. Matching Line Features by GMM

The line feature matching problem can be converted to solve a Gaussian mixture model (GMM) [18]. The essence of GMM is a weighted composition of several normal probability density functions with different parameters. Through the previous procedures, linear features of registration image pairs have been obtained. Suppose that the line segments in the reference image are centroids of GMM, and the line segments in the image to be registered can serve as observed data. The corresponding relation of the line segments in the image pair can be regarded as hidden variables, so as to estimate transform parameters for rough matching.

Firstly, consider an arbitrary image to be registered *J* and its reference image *I*. Let *N* be the number of line segments in *I*, and *M* is the number of line features in *J*. Let *X =* {*x*_1_*, x*_2_,*…*, *x_N_*} for the line segment set detected from *I*; *Y =* {*y*_1_, *y*_2_,*…, y_M_*} for the line segment set detected from *J*; and ***P*** = {*P_mn_*}_1__≤_*_m_*_≤_*_M_*_, 1_*_≤__n__≤__N+_*_1_ is a response matrix, which is composed of hidden variables of GMM [19,20]. If *n* ≤ *N +* 1, then *P_mn_* is the probability that the line segment *y_m_* is related to *x_n_*, and if *n = N +* 1, then *y_m_* is an outlier. The problem of line segment matching can be expressed as a likelihood equation below [21].

(3) $$L(\Theta )=\ln\prod_{m=1}^{M}p(y_{m})$$

where *p*(*y_m_*) is the marginal probability distribution of *y_m_* in a GMM, and *Θ* is the parameter set of an affine transform model. The transform of *x_n_* under the model parameter set *Θ* can be denoted as *T* = (*x_n_*, *Θ*). Since the relationship between line segments is unknown, it is difficult to maximize *L* directly. Therefore, this problem can be resorted to the expectation maximization (EM) algorithm to realize the optimization of *L* through iteration [22].

### 2.2. Fine Matching

According to line matched pairs, the parameters of the affine transform model of the image can be estimated. However, owing to large differences of view angles, there are some mismatches in the line matching results. They will affect the estimation of transform parameters. Hence, it is essential to conduct fine matching based on point features. Specifically, this contains the following steps: point feature extraction, point feature matching, and mismatched point elimination.

#### 2.2.1. Extraction and Representation of Point Features

Because rough matching has been fulfilled, the simple Harris operator is chosen to detect feature points [23] and the popular SIFT operator is utilized to describe point features [24,25]. Corner detection is executed both on the reference image and on the transformed image according to the rough registration model [26].

#### 2.2.2. Point Feature Matching

At first, a K-D (K-Dimensional) tree of point features is constructed [27], and all data are divided into left and a right subtrees according to their spatial location. Then, the same operations are conducted on the data in the subtrees until all points have been processed. In the processes of division, it is necessary to maintain the data balance between the left and right subtrees as far as possible. Otherwise, the search efficiencies will be reduced.

Then, matched point pairs are searched for by using the BBF (Best Bin First) strategy [28]. The BBF strategy is a search algorithm for the K-D tree structure and outperforms the K-D tree search algorithm in high-dimensional features. The BBF algorithm improves search efficiency by establishing a priority queue and by setting the maximum number of backtracking and maximum running time. It pushes points that may be backtracked into the queue and ranks them according to their distances from the hyperplane of a search point. The closest point possesses the highest priority. Then, every point is traversed by their priority until the sequence becomes empty.

Meanwhile, the first/second-nearest neighbor ratio method is applied for similarity comparison of the point features. Accordingly, it is required to find two points in the image to be registered that are firstly/secondly closest to a search point in the reference image. Their distances are denoted as *Dis*1 and *Dis*2, respectively. Then, the value of *Dis*1/*Dis*2 is compared with a given threshold. When the ratio is less than the threshold, it can be believed that the current point pair may be a matched pair [29].

#### 2.2.3. Elimination of Mismatches

Taking large differences of view angles into consideration, projection transforms are adopted in fine matching stages. Mismatches are eliminated in order to yield optimal parameters for a projection transform matrix by the RANSAC algorithm [30,31]. Finally, stitched images are generated by a bilinear interpolation method.

## 3. Experimental Results

The hardware environment mainly includes an Intel Core i5-8250U processor at 1.80 Hz, with 4.00 GB RAM. The operating system is 64-bit Windows 10, and the programming software is MATLAB R2014a.

### 3.1. Rough Matching Test

**Figure 4:**
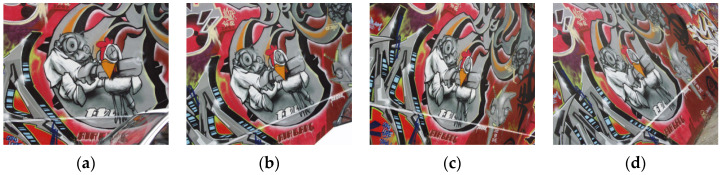
The multi-angle images about a same scene applied in the rough matching test: (**a**) the 0° image; (**b**) the 10° image; (**c**) the 20° image; (**d**) the 50° image.

**Figure 5:**
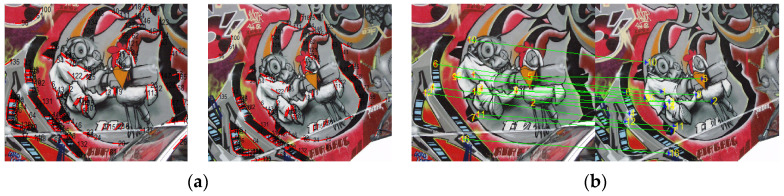
The matching results for the first pair of images: (**a**) the line matching result; (**b**) the point matching result.

In order to verify the effectiveness of our line matching algorithm, a classic group of multi-angle images about a same scene in the reference [32] is applied in our rough matching test. The angles of these experimental images are 0°, 10°, 20°, and 50°, as shown in Figure 4. There are three image pairs designed for the matching test. The first pair is 0° and 10° images, the second pair is 0° and 20° images, and the third pair is 0° and 50° images. The 0° image is the reference image, and our line matching method is compared with a popular point matching algorithm, i.e., SIFT, in this experiment. Figure 5 displays the matching results for the first pair of images.

The quantitative indicator comparison of the two matching methods is presented in Table 1, which includes the number of matched pairs (NoP) and accuracy. It can be seen from Table 1 that for multi-angle images, the line matching method proposed in this work is more reliable than the point matching method. As the difference of view angles increases, matched feature pairs obtained by the two methods decrease accordingly. The NoP of our line matching method is much higher than that of the point matching method. However, the point matching method is outstanding at accuracy. Thus, it is manifested that combining the line and point matching in multi-angle optical image registration is reasonable for improving registration results.

### 3.2. Registration Results and Analysis

**Figure 6:**
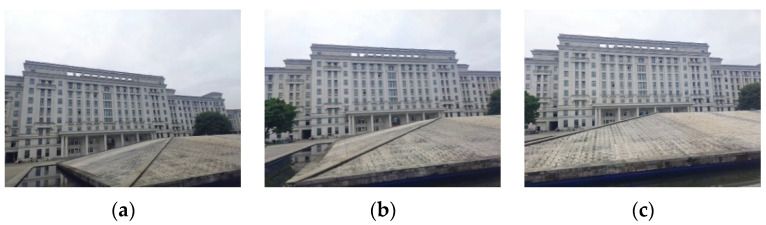
The original multi-angle images of the first scene: (**a**) the 0° image; (**b**) the 15° image; (**c**) the 35° image.

The multi-angle registration method proposed in this work is verified in this section. The multi-angle optical images of ground scenes we used are taken by a common digital camera. The multi-angle images from the first scene are given in Figure 6.

**Figure 7:**
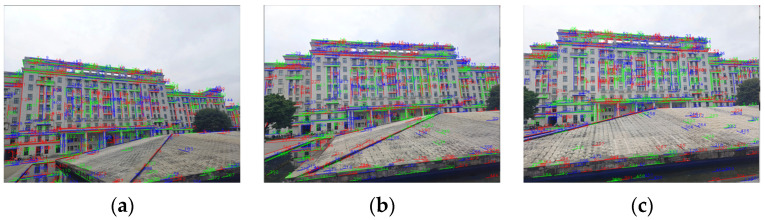
The extracted line features from the multi-angle images of the first scene: (**a**) the 0° image; (**b**) the 15° image; (**c**) the 35° image.

The sizes of the three images in Figure 6 are all 800 × 600 pixels. Similarly, the 0° image serves as the reference image. Figure 7 exhibits the results of the line feature extraction by the LSD algorithm. The numbers of the line segments in each image of the first scene are 432, 466, and 464, respectively.

**Figure 8:**
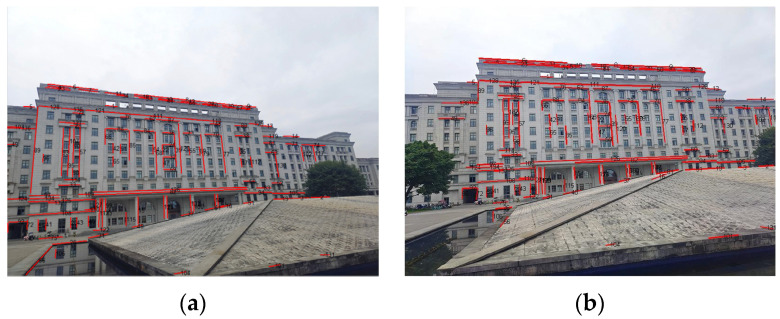
The rough matching results of the first scene: (**a**) the 0° image; (**b**) the 15° image.

**Figure 9:**
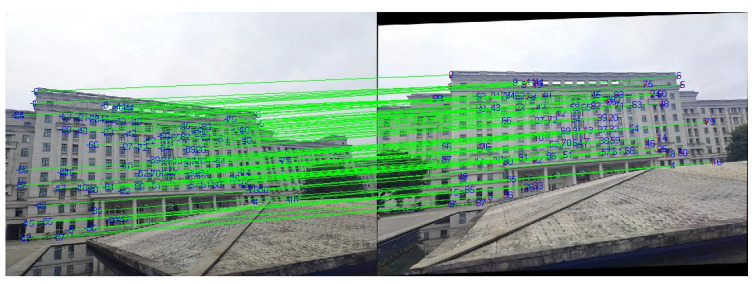
The fine matching results for the 0°and 15° images of the first scene.

After rough matching, 137 matched line pairs are obtained from the 0° image and 15° image, as shown in Figure 8. Then, the 15° image in Figure 6 is transformed by a corresponding affine model. On this basis, fine matching is carried out and the results are shown in Figure 9.

**Figure 10:**
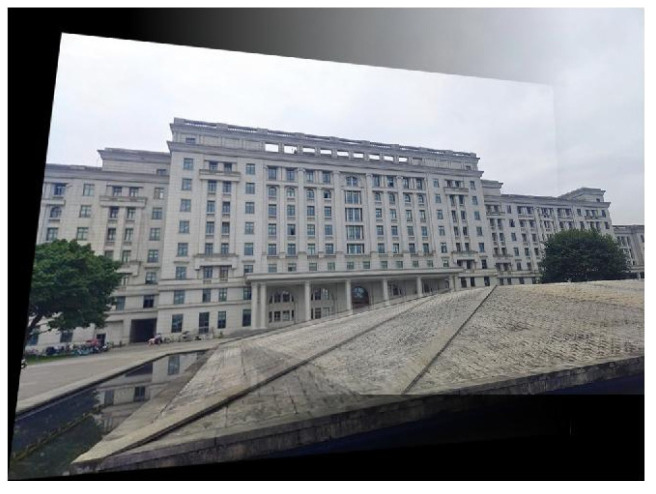
The stitched image generated by using ***M****_ab_*.

In Figure 9, there are 85 matched point pairs, and the obtained projection transform matrix ***M****_ab_* is specified by Equation (4). According to this transform matrix, the 0°and 15° images are registered and stitched together. The final stitched result is shown in Figure 10.

(4) $$M_{ab}=\left[\begin{array}{ccc} 0.8464 & 0.0939 & 88.6506\\ -0.1148 & 0.9715 & -20.2125\\ 0.0000 & 0.0000 & 1.0000 \end{array}\right]$$

**Figure 11:**
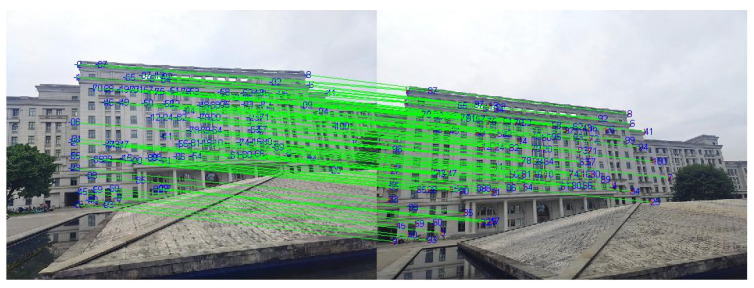
The direct point matching results for the 0°and 15° image of the first scene.

Meanwhile, the 0°and 15° images of the first scene are also matched through the point-based SIFT method. As can be seen in Figure 11, there are 100 matched point pairs, and the obtained projection transform matrix is shown in Equation (5).

(5) $$M_{\mathrm{point}}=\left[\begin{array}{ccc} 0.8414 & 0.0979 & 87.9872\\ -0.1135 & 1.0276 & -50.5910\\ -0.0003 & 0.0002 & 1.0000 \end{array}\right]$$

**Figure 12:**
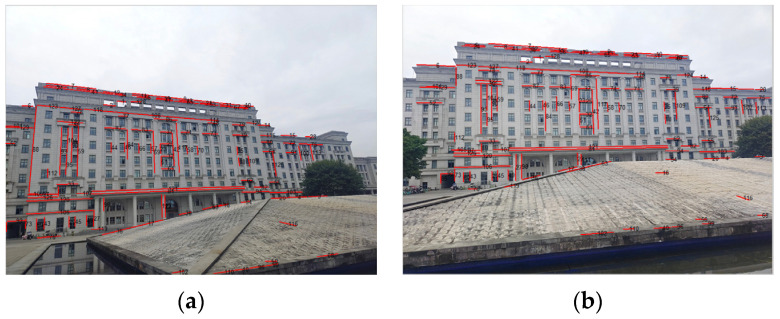
The rough matching results for the 0°and 35° images of the first scene: (**a**) the 0° image; (**b**) the 35° image.

The rough matching results for the 0°and 35° images of the first scene are presented in Figure 12 and, in total, there are 129 matched line pairs generated by the proposed method.

**Figure 13:**
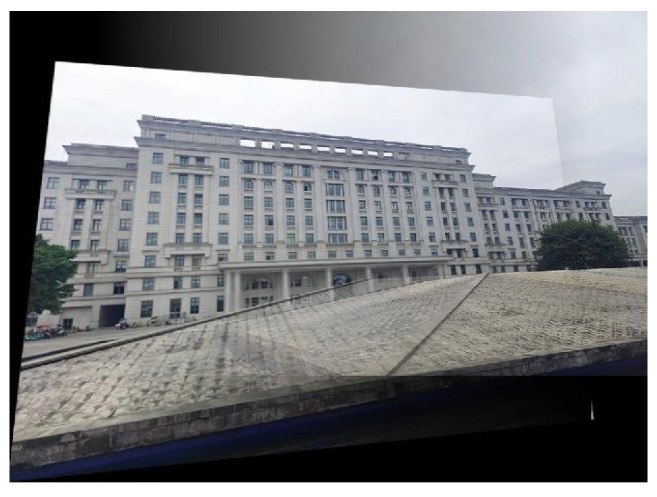
The stitched image generated by using ***M****_ac_*.

Then, the affine transform is performed on the 35° image in Figure 6. Similarly, there are 70 pairs of matched point pairs yielded by fine matching. The final projection transform matrix ***M****_ac_* is expressed as Equation (5), and the stitched image is displayed in Figure 13.

(6) $$M_{\mathrm{ac}}=\left[\begin{array}{ccc} 0.8920 & 0.0797 & 50.3169\\ -0.1061 & 1.0623 & -81.7635\\ -0.0003 & 0.0003 & 1.0000 \end{array}\right]$$

In addition, the proposed registration algorithm combining point and line features are quantitatively compared with the SIFT algorithm and the detailed results are listed in Table 2. We also compare the proposed method with three classical point-based registration methods, including BRISK (Binary Robust Invariant Scalable Keypoints) [33], KAZE [34], and SURF (Speed-Up Robust Features) [35].

In Table 2, it can be noticed that the number of matched features decreases with the increase of the view angle differences. Although SIFT and SURF can obtain more matching point pairs than the method proposed in this work, their correct matching rate is not as completely good as our method. These results indicate that our feature extraction strategy is more reliable than the other algorithms in terms of correct matching. Moreover, the RMSE of our registration algorithm is also superior to the other algorithm. Since adding the line matching process, our algorithm has no significant advantages in speed. However, its time consumption is still acceptable in general cases.

**Figure 14:**
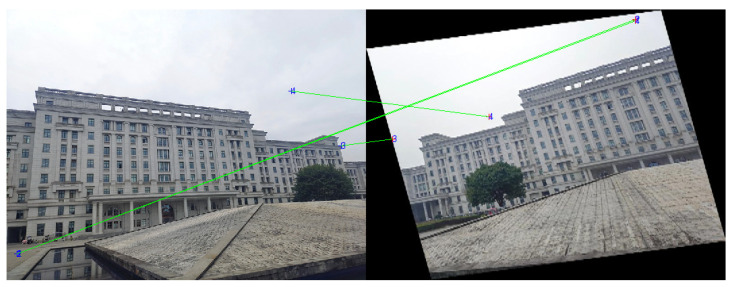
An example of matching failure for the 0°and 50° images of the first scene.

In addition, it is also found in experiments that our registration strategy can provide good performances for images with angle differences from 0° to 45°. If an angle difference is less than 35°, its RMSE can maintain within 1.10 to 1.15. When the angle difference reaches 45°, the RMSE value of our method rises to 1.4149, and its accuracy is still slightly higher than those of point matching methods. However, with the angle difference increasing continuously, registration accuracy will decline. The reason is that it is difficult to successfully find a matched line or point features in this case. When the angle difference exceeds 45°, the transform angle of an affine model generated by line matching will become too large, and thus, point features cannot be extracted during fine matching. This situation will result in matching failure, as illustrated in Figure 14.

**Figure 15:**
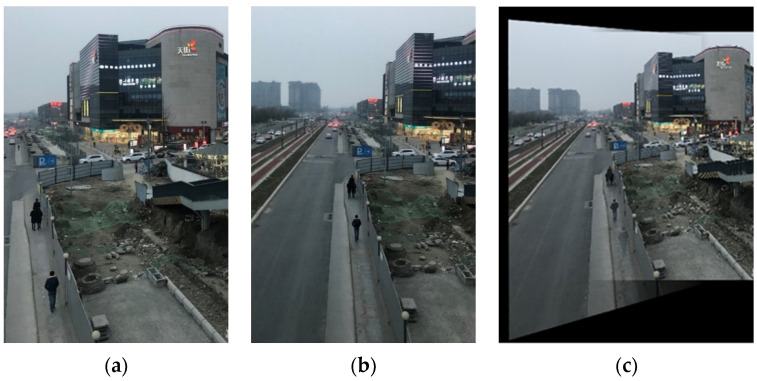
The experimental images of the second scene: (**a**) the reference image; (**b**) the image to be registered; (**c**) the stitched image.

The experimental images of the second scene are both of 800 × 1200 pixels with relatively few overlapping regions. There are 129 matched line pairs obtained by rough matching. Then, an affine transform is performed on the image to be registered in Figure 15 in order to accomplish fine matching. Figure 15c is the stitched image of the second scene. The other algorithms are also applied to the images of the second scene and their comparison results with the proposed algorithm of this work are listed in Table 3.

According to the results in Table 3, it can be proved that, for multi-angle images, the registration accuracy of our method based on both line and point features is also higher than those of the other excellent algorithms. Similarly, although SIFT, KAZE, and SURF can obtain more matching point pairs than the proposed method, their correct matching rates are, in fact, not superior to our method. Thus, the reliability of our method is also verified. If there are no strict time requirements, the introduction of line features can generate matched point pairs more effectively, so as to enhance the registration accuracy for multi-angle images.

## 4. Conclusions

Focused on multi-angle optical images of ground scenes, this work combines line features with point features to improve the quality of image registration. It utilizes line features to realize rough matching. Furthermore, in order to achieve more accurate registration, it makes use of the point features of images in fine matching. To begin with, linear features are extracted through the LSD algorithm. An iteration method is designed based on GMMs to match extracted linear segments and optimize parameters of affine transform models. Lastly, the reference image and the roughly-transformed image are finely registered by using point features. The experiment results indicate that the registration strategy proposed in this work can register multi-angle images effectively without any artificial intervention or sample training, and it outperforms the mere point-based registration methods in registration accuracy. Since it has no special requirements for hardware and software platforms, our method also has obvious advantages in adaptability. It can be easily realized on small-size or mobile computing devices in order to satisfy practical application requirements.

Due to the combination of the detection and matching of different features, our proposed registration algorithm has no advantages in time. Therefore, it is also a critical issue to improve calculation efficiency in further research. Meanwhile, the multi-angle registration in this work is carried out in two-dimensional cases. It cannot cope with those images with quite large divergences of view angles. Hence, it is challenging to fulfill the matching in stereo space based on image features and to improve adaptabilities to more complex scenes in future algorithm design. Moreover, deep learning technology can also be exploited to improve registration effects for multi-angle images in subsequent research because of its strong feature extraction capability.

## Figures and Tables

**Table 1:** The quantitative comparison of two matching methods.

Methods	The First Pair		The Second Pair		The Third Pair	
	NoP	Accuracy of Pairs	NoP	Correct Matching Rate	NoP	Accuracy of Pairs
line matching	152	92.3%	108	90.2%	50	84%
point matching	16	100%	16	100%	7	100%

**Table 2:** The registration result comparison of the first scene.

Images	Methods	NoP (Lines)	NoP (Points)	Correct Matching Rate	RMSE	Time (s)
0° and 15°	SIFT	/	100	98%	1.1892	4.370
	BRISK	/	58	100%	1.1953	2.136
	KAZE	/	89	98%	1.2137	4.678
	SURF	/	122	98.3%	1.2059	3.733
	Ours	137	85	100%	1.1161	8.527
0° and 35°	SIFT	/	73	100%	1.1676	4.292
	BRISK	/	52	99%	1.1715	2.032
	KAZE	/	64	100%	1.1708	4.489
	SURF	/	95	98%	1.1803	3.376
	Ours	129	70	100%	1.1389	8.378

**Table 3:** The registration result comparison of the second scene.

Methods	NoP (Lines)	NoP (Points)	Correct Matching Rate	RMSE	Time (s)
SIFT	/	121	97.5%	1.5151	11.934
BRISK	/	78	96.8%	1.5619	5.466
KAZE	/	116	96.3%	1.5722	14.659
SURF	/	134	97.8%	1.5312	7.904
Ours	129	112	98.2%	1.4777	48.315

## Data Availability

The images and source codes involved in this work are available from the authors upon reasonable requests.
